# Rural Health Curriculum in Bachelor of Science in Nursing Programs: A Narrative Review

**DOI:** 10.1111/jrh.70200

**Published:** 2026-08-05

**Authors:** Kyanna Harris, Hilary N. Touchett, Alexandra Caloudas, Kelley Arredondo

**Affiliations:** ^1^ Houston VA HSR&D Center for Innovations in Quality, Effectiveness, and Safety Michael E. DeBakey VA Medical Center Houston Texas USA; ^2^ Department of Medicine Baylor College of Medicine Houston Texas USA; ^3^ VA South Central Mental Illness Research, Education Clinical Center, a Virtual Center Houston Texas USA; ^4^ Menninger Department of Psychiatry and Behavioral Sciences Baylor College of Medicine Houston Texas USA; ^5^ VHA Office of Rural Health's Veterans Resource Center in Salt Lake City Salt Lake City Utah USA; ^6^ VHA Office of Rural Health's Veterans Resource Center in White River Junction White River Junction Vermont USA

**Keywords:** curriculum, narrative review, nursing education, rural health, workforce development

## Abstract

**Purpose:**

Rural communities face persistent health disparities and ongoing healthcare workforce shortages, especially in nursing. Despite national attention on strengthening the rural nursing pipeline, little synthesis exists on how rural health is integrated into nursing curricula. This narrative review provides an overview of the current landscape of rural health content within Bachelor of Science in Nursing (BSN) programs in the United States.

**Methods:**

A comprehensive search of peer‐reviewed and institutional sources was conducted using CINAHL, PubMed, ERIC, Scopus, and the Commission on Collegiate Nursing Education (CCNE) database. U.S.‐based BSN programs with explicit rural health training or learning components were included. Data were extracted and synthesized using a narrative approach to identify recurring themes and institutional variations.

**Findings:**

Sixty‐three nursing programs met our inclusion criteria; however, many had various courses or initiatives for a total of 93 courses/initiatives. The majority of courses/initiatives were community‐focused (*n* = 59), followed by underserved communities (*n* = 37). Few BSN programs had explicit rural health content (*n* = 20) that incorporated rural‐specific courses or structured clinical experiences addressing rural practice.

**Conclusions:**

Rural health remains underrepresented in BSN curricula. Findings underscore the need for systematic integration of rural concepts through coursework, simulation, and partnerships with rural healthcare facilities to strengthen the rural nursing workforce.

## 1 Introduction

Nurses play a central role in delivering healthcare to rural populations in the United States, where residents experience persistent health disparities and reduced access to care [1, 2, 3]. Most recent census data reports that 19.3% of the U.S. population lives in rural areas [4]. Rural residents are typically older, have lower incomes, experience higher rates of chronic disease, and have worse nonmedical factors (e.g., economic stability, educational quality) than their urban counterparts [5]. These challenges are compounded by longstanding rural healthcare workforce shortages, with 64% of nonmetropolitan counties designated as primary care Health Professional Shortage Areas, compared to 41% of metropolitan counties [6]. Workforce shortages and related financial pressures have also contributed to the closure and loss of essential service reductions in rural healthcare facilities, such as 146 rural hospital closures between 2005 and 2023, and more than 25% of rural hospitals losing obstetric services across the United States [7, 8]. Ultimately, these rural hospital closures and loss of services have forced rural residents to travel farther for essential care, creating additional barriers to their healthcare access [6].

Preparing a nursing workforce capable of meeting the needs of rural populations is, therefore, a priority. Rural nursing has emerged as a distinct area of practice demanding broad clinical competence, autonomy, adaptability, and the ability to provide comprehensive care across the lifespan [9, 10]. In addition to strong clinical skills, rural nurses must also effectively engage with local communities, develop partnerships, and respond to the unique cultural, social, and environmental contexts that influence health in rural settings. These competencies extend beyond those typically emphasized in traditional nursing curricula and require educational experiences that prepare graduates for the realities of rural practice. Consequences of lacking rural‐specific training may lead to higher turnover and retention issues and decreased quality of care [11].

Despite the importance of rural nursing, rural health‐related content remains inconsistently integrated into Bachelor of Science in Nursing (BSN) programs. Prior research suggests that out of 237 BSN programs, fewer than half include any rural health content, but little is known about the content characteristics [12]. Curriculum that includes a rural‐related focus such as community engagement, population health, underserved communities, and social determinants of health (SDOH) is critical for preparing nurses to provide context‐sensitive care.

Synthesizing this literature is further complicated by the absence of a single, universally accepted definition of “rural.” Federal agencies, researchers, and healthcare organizations use multiple classification systems based on geography, population size, commuting patterns, and healthcare access [13]. Consequently, educational programs may define rural practice differently or use varying criteria to describe rural experiences. Rather than applying a single external definition of rural, this review sought to characterize how rural health‐related content is represented within BSN curricula. Therefore, we did not define rural but instead sought to identify content that was labeled as rural or rural‐related, such as community or population health.

A comprehensive understanding of how rural health is currently incorporated into BSN education is needed to identify curricular gaps and inform future educational strategies that strengthen the rural nursing workforce. Therefore, the purpose of this review was to examine the integration of rural health‐related content within U.S. BSN programs.

Specifically, we sought to answer the following questions:

1. To what extent do BSN programs incorporate rural health‐related content in their curriculum?
2. What are the characteristics (e.g., program type, program length, population focus) of rural health‐related content in BSN programs?

## 2 Methods

### 2.1 Study Design

We conducted a narrative review of both published and gray literature on rural health‐related content in BSN programs. A narrative review is particularly appropriate for gaining a comprehensive understanding of a subject, synthesizing, and identifying areas for further research [14].

### 2.2 Search Strategy

For the published literature, we searched PubMed, CINAHL, ERIC, Scopus, and Google Scholar between March and May 2026 using the following search terms: *(“BSN program” OR “Bachelor of Science in Nursing” OR “nursing program”) AND (“community health” OR “population health” OR “rural healthcare” OR “rural health”)*.

For the gray literature, BSN programs were searched using both keyword‐based internet searches and the Commission on Collegiate Nursing Education (CCNE) database between April 2025 and late July 2025 [15]. The CCNE database contains information on all CCNE‐accredited programs from 1997 to the present. The website provides a list of all Baccalaureate nursing programs nationwide (*n* = 844) organized by alphabetical letter. To search a representative sample of Baccalaureate nursing programs, we reviewed over half of the nursing programs by randomly selecting letters that, on average, had 35 programs each (A, B, C, E, F, G, K, L, M, O, P, R), for a total of 430 programs reviewed (430/844; 51%).

We also conducted internet searches using Google for BSN programs with search terms such as college rural nursing course, rural nursing course, college BSN program + rural, BSN program + rural, rural nursing courses U.S., NURS rural, BSN program + NURS rural, BSN programs with rural in the course title, and Locate BSN programs that include rural in course titles.

### 2.3 BSN Program Inclusion Criteria

BSN programs in the literature and gray literature included in this review were (a) published in English, (b) based in the United States, (c) a BSN‐level program, (d) mentioned community health, population health, or rural healthcare in the curriculum, training elements, or courses offered, and for the published literature, (e) published between 2018 and 2025.

### 2.4 BSN Program Selection

#### 2.4.1 Published Literature

The articles identified from the search terms across PubMed, CINAHL, ERIC, Scopus, and Google Scholar were imported into Covidence [16], and each title and abstract was reviewed by two independent screeners (KH & KA). Those selected for full‐text review were also reviewed by two independent reviewers (KH & KA) to determine whether to include the manuscript in the review. All conflicts were discussed by the two coders until consensus was reached.

#### 2.4.2 Gray Literature

BSN programs in each of the selected letters (A, B, C, E, F, G, K, L, M, O, P, R) on the CCNE website were reviewed to determine if the program met the inclusion criteria. BSN programs were reviewed by two screeners (KH & KA) to determine whether they met the inclusion criteria. Furthermore, BSN programs that were identified through a Google internet search were also reviewed to determine if the program met the inclusion criteria.

### 2.5 Data Extraction

Information from BSN programs that met the inclusion criteria were extracted by two coders (KH & KA). Discrepancies were discussed among the two coders until consensus was reached. Data for each BSN program were entered into an excel sheet which included: rural‐related program or course title, program type (i.e., clinical experience, didactic, experiential learning, simulation, case study, course), program or course length, delivery method (i.e., in‐person, online, hybrid), population focus (i.e., general community, underserved communities, population health, rural, social determinants or health, Indigenous/Tribal).

## 3 Results

The literature review yielded 26 BSN programs, and the gray literature produced 37 BSN programs that met the inclusion criteria for a total of 63 BSN programs. Some BSN programs had more than one rural‐related course/initiative, yielding a total of 93 courses/initiatives. The results will depict the characteristics of the 93 courses/initiatives (see Supplementary Material 1 for a list of all courses/initiatives).

The components of the courses/initiatives were coded by type (i.e., didactic, course, clinical experience, experiential, simulation experience, case study), with many courses/initiatives having multiple components. Most of the courses/initiatives had a didactic component (*n* = 69), followed by clinical experience (*n* = 32), and experiential (*n* = 22), with some having a simulation experience (*n* = 4) or presenting learners with a case study (*n* = 3). The majority of courses/initiatives offered a fully in‐person option (*n* = 71), while others offered a fully online option (*n* = 41), and some were hybrid (*n* = 13). Course/initiative length varied, with the longest lasting 2 years (*n* = 1) and the shortest consisting of 2–3 days (*n* = 1; see Table 1 for all course/initiative lengths).

**TABLE 1:** Course/initiatives by length of time.

Course/initiative length	Frequency
16 weeks	69
1 year	11
1 day	4
2−3 days	1
10 weeks	1
15 months	1
2 years	1
N/A	5

Courses/initiatives were also coded by the population they were focused on (e.g., community [general], underserved, rural). The majority of courses/initiatives focused on general community health (*n* = 59), followed by underserved communities (*n* = 37; see Table 2).

**TABLE 2:** Courses/initiatives by population focus.

Population focus	Frequency
Community (general)	59
Underserved communities	37
Population health	26
Rural	20
Social determinants of health	13
Indigenous/Tribal	4

Furthermore, the 93 courses/initiatives that met the inclusion criteria were in 31 different states, with the highest number in Texas (*n* = 7) and California (*n* = 6; see Table 3).

**TABLE 3:** Number of rural‐related courses/initiatives by state.

State	Frequency
Texas	7
California	6
Virginia	5
Tennessee	5
Minnesota	5
Georgia	4
North Dakota	4
South Dakota	4
Washington	4
Maine	3
Wisconsin	3
Florida	3
Kentucky	3
Michigan	3
Montana	3
South Carolina	3
Nevada	3
Vermont	3
Arizona	2
Illinois	2
Massachusetts	2
West Virginia	2
Mississippi	2
Wyoming	2
Colorado	1
New Mexico	1
New York	1
North Carolina	1
Ohio	1
Oregon	1
Pennsylvania	1

*Note*: One course/initiative did not specify its location, and another course/initiative was offered nationally.

## 4 Discussion

This narrative review provides one of the first national examinations of how rural health‐related content is incorporated within U.S. BSN curricula by synthesizing both published and gray literature. Rather than limiting our review to programs explicitly labeled as “rural,” we intentionally examined broader curricula content that included community health, population health, underserved populations, and social determinants of health because many educational experiences that prepare students for rural practice are not explicitly described in rural terminology. Our findings suggest that although many BSN programs provide educational experiences relevant to rural practice, explicit identification of rural curricular priority remains inconsistent. Consequently, it remains difficult to determine the extent to which nursing graduates are intentionally prepared to address the unique healthcare needs of rural communities.

### 4.1 Rural Health Preparation Often Occurs Indirectly

The majority of the 93 identified courses and initiatives focused on general community health (*n* = 59), followed by underserved populations (*n* = 37), while substantially fewer explicitly identified rural populations as their primary focus (*n* = 20). These findings suggest that rural health is frequently encompassed within broader community and population health frameworks rather than being treated as a distinct area of nursing education. Although these broader curricula likely provide competencies that are applicable to rural practice, including community engagement, health promotion, and addressing SDOH, they may not fully prepare students for the additional realities of rural nursing.

Since rural nurses frequently practice with greater autonomy, broader clinical responsibilities, and fewer specialty resources [9, 10], they must also build long‐term relationships within closely connected communities and adapt care to local cultural, social, and environmental contexts. These competencies extend beyond traditional community health education and require intentional discussion of how healthcare delivery differs in rural environments. Without explicitly framing these concepts within rural practice, students may complete community health coursework without recognizing how their knowledge and skills translate to rural healthcare settings.

The lack of consistent rural terminology across curricula also reflects the broader challenge of defining “rural.” Federal agencies and researchers use multiple classification systems based on geography, population density, commuting patterns, and healthcare access, resulting in considerable variability in how rural communities are identified [13]. Consequently, nursing programs may intentionally incorporate rural experiences while describing them using broader language such as community health, medically underserved populations, or population health. At the same time, the lack of consistent terminology makes it difficult to identify best practices and evaluate the degree to which rural competencies are intentionally incorporated across nursing programs.

### 4.2 Variability in Educational Approaches to Rural Health

Our review also demonstrates considerable variation in how rural health‐related content is delivered. Didactic instruction was the most common educational approach (*n* = 69), followed by clinical experiences (*n* = 32) and experiential learning opportunities (*n* = 22), with relatively few programs incorporating simulation or case‐based learning. While classroom instruction provides an important foundation, the predominance of didactic approaches suggests that many students may primarily learn about rural health rather than actively develop the clinical reasoning, adaptability, and resourcefulness required in rural practice.

Experiential and place‐based learning are particularly valuable in rural nursing education because they may allow students to experience resource‐limited environments, develop relationships with rural communities, and better understand the contextual factors that influence health and healthcare delivery. Programs that incorporated rural clinical placements, community partnerships, longitudinal experiences, or other immersive educational models appear well positioned to enhance students’ understanding of rural practice while fostering interest in rural careers. Expanding these opportunities may better prepare graduates for the realities of rural nursing and strengthen the future rural workforce.

Similarly, course length, instructional format, and program structure varied considerably across institutions, suggesting there is currently no standard approach for integrating rural health into BSN education. This variability likely reflects differences in institutional missions, faculty expertise, geographic location, and access to rural clinical partners. While flexibility allows programs to tailor curricula to local needs, greater consistency regarding core rural health competencies may ensure that all graduates possess a foundational understanding of rural healthcare regardless of where they receive their nursing education.

### 4.3 Implications for Nursing Education

Preparing a nursing workforce capable of addressing rural health disparities requires more than offering community health courses or isolated rural clinical experiences. Nursing programs should consider intentionally integrating rural perspectives throughout the curriculum by connecting community health concepts to rural contexts, incorporating rural case studies, highlighting rural health disparities, and providing opportunities for students to engage directly with rural communities whenever feasible. This is an important approach for individuals who need to develop skills to help a specific population [17]. Embedding rural concepts longitudinally throughout the curriculum may help students appreciate the distinctive characteristics of rural nursing while reinforcing competencies that are applicable across healthcare settings.

To strengthen the rural nursing pipeline, BSN programs must consider moving beyond generalized population health content to incorporate place‐specific, context‐sensitive curricula. This approach includes offering dedicated rural health courses, integrating rural examples across core content areas, and expanding clinical placement opportunities in rural settings. Moreover, strategic collaboration between academic institutions and rural healthcare facilities can foster co‐created community‐embedded learning experiences that enhance both student competence and rural workforce retention [18].

Partnerships between academic institutions and rural healthcare organizations may further strengthen this preparation. Community‐engaged educational models, longitudinal rural clinical experiences, and collaborative relationships with rural health systems have the potential to improve student understanding of rural practice while simultaneously strengthening local workforce pipelines [18]. Such partnerships may also help address recruitment and retention challenges by increasing students’ familiarity with rural communities and fostering interest in rural careers [19, 20].

Institutional commitment is equally important. Programs that explicitly recognize rural health within curricular objectives, program descriptions, or strategic priorities may be better positioned to develop sustainable educational experiences and cultivate faculty expertise in rural health. Although rural content may not need a standalone course, clearly embedding rural competencies throughout the program's curriculum and experiential elements may increase rural visibility and ensure intentional preparation for rural practice.

### 4.4 Strengths and Limitations

This review has several strengths. By combining published literature with gray literature from accredited BSN programs, we provide a comprehensive overview of how rural health‐related content is represented within nursing education across the United States. Inclusion of institutional curricula in addition to published studies allowed us to examine educational practices beyond those reported in the literature and identify a broader range of curricular approaches.

However, some limitations must be acknowledged. First, curriculum analysis relied on publicly available information, which may not fully reflect internal or informal rural content. Because this review intentionally included community health, population health, underserved populations, and related content, not every identified course or initiative was exclusively focused on rural healthcare. This broader approach was necessary because many programs appear to prepare students for rural practice without explicitly labeling those educational experiences as rural. Third, only approximately half of CCNE‐accredited BSN programs were included in the gray literature review, although programs were randomly sampled to provide broad geographic representation. Finally, we included programs as long as they resulted in a BSN but did not distinguish between RN‐BSN, accelerated BSN programs, and traditional BSN programs.

### 4.5 Future Directions

Future research should move beyond documenting whether rural health‐related content exists and examine how it is implemented, experienced, and evaluated. Studies exploring faculty perspectives, student experiences, and institutional decision‐making could identify barriers and facilitators to integrating rural health throughout nursing curricula. Longitudinal research is also needed to determine which educational approaches, including rural clinical placements, simulation, community partnerships, and immersive rural experiences, most effectively improve rural competencies, influence career intentions, and ultimately contribute to recruitment and retention within the rural nursing workforce.

Adding accreditation standards, procedures, and guidelines for core rural nursing competencies and encouraging more consistent reporting of rural educational content would facilitate future curriculum evaluation while allowing programs flexibility in how those competencies are achieved.

## 5 Conclusions

Addressing rural health disparities requires a nursing workforce that is not only clinically prepared but also contextually informed and motivated to serve in rural communities. This review supports that, while innovative educational models and rural‐focused curricula exist, their implementation across BSN programs remains limited. Increasing the visibility of rural health within BSN curricula, expanding experiential learning opportunities, strengthening partnerships with rural communities, and intentionally integrating rural competencies throughout nursing education may better prepare graduates to meet the complex healthcare needs of rural populations and contribute to a stronger and more sustainable rural nursing workforce.

## Funding

This work was partially funded by the Department of Veterans Affairs, Veterans Health Administration, Office of Rural Health, NOMAD #PROFY‐010172 and supported by using resources and facilities at the Houston VA HSR&D Center for Innovations in Quality, Effectiveness and Safety (CIN13‐413) and the South‐Central Mental Illness Research, Education and Clinical Center. The views expressed are those of the authors and not necessarily those of the Department of Veterans Affairs, the United States Government, or Baylor College of Medicine.

## Disclosure

The authors have nothing to report.

## Conflicts of Interest

The authors declare no conflicts of interest.

## Supporting information




**Supporting File 1**: jrh70200‐sup‐0001‐SuppMat.xlsx

## Data Availability

The data that support the findings of this study are available from the corresponding author upon reasonable request.
